# Blood-Brain Barrier Disruption by Lipopolysaccharide and Sepsis-Associated Encephalopathy

**DOI:** 10.3389/fcimb.2021.768108

**Published:** 2021-11-04

**Authors:** Xiaoyao Peng, Zhixuan Luo, Shuang He, Luhua Zhang, Ying Li

**Affiliations:** ^1^Department of Clinical Medicine, School of Clinical Medicine, Southwest Medical University, Luzhou, China; ^2^Department of Pathogenic Biology, School of Basic Medical Sciences, Southwest Medical University, Luzhou, China; ^3^Department of Immunology, School of Basic Medical Sciences, Southwest Medical University, Luzhou, China

**Keywords:** blood-brain barrier, lipopolysaccharide, sepsis-associated encephalopathy, central nervous system, inflammation, oxidative stress

## Abstract

As a complex multicellular structure of the vascular system at the central nervous system (CNS), the blood-brain barrier (BBB) separates the CNS from the system circulation and regulates the influx and efflux of substances to maintain the steady-state environment of the CNS. Lipopolysaccharide (LPS), the cell wall component of Gram-negative bacteria, can damage the barrier function of BBB and further promote the occurrence and development of sepsis-associated encephalopathy (SAE). Here, we conduct a literature review of the direct and indirect damage mechanisms of LPS to BBB and the relationship between these processes and SAE. We believe that after LPS destroys BBB, a large number of inflammatory factors and neurotoxins will enter and damage the brain tissue, which will activate brain immune cells to mediate inflammatory response and in turn further destroys BBB. This vicious circle will ultimately lead to the progression of SAE. Finally, we present a succinct overview of the treatment of SAE by restoring the BBB barrier function and summarize novel opportunities in controlling the progression of SAE by targeting the BBB.

## Introduction

All organisms with a well-developed central nervous system (CNS) have a blood-brain barrier (BBB). The CNS has always been in a dynamic equilibrium environment that is significantly different from the peripheral environment and this is mainly determined by the barrier function of the BBB (Obermeier et al., 2013). The BBB is mainly composed of vascular endothelial cells (ECs) and basement membrane (BM), and there are also pericytes and astrocyte end-feet covering them, which together constitute the neurovascular unit (NVU) to maintain the function of BBB (Armulik et al., 2010; Yao et al., 2014; Kutuzov et al., 2018; Nikolakopoulou et al., 2019; Huang et al., 2020b; Kaplan et al., 2020; Yue et al., 2020).

In addition, neurons and microglial cells, as components of the NVU, can also directly or indirectly affect the development and functions of the BBB (da Fonseca et al., 2014; Xu et al., 2017; Haruwaka et al., 2019; Su et al., 2020; Zhang et al., 2020). As a protective barrier at the interface of the brain and the peripheral environment, BBB successfully shields the CNS from toxic substances circulating in the blood by strictly controlling the material exchange between blood and brain tissue (Liebner et al., 2018). And, as a dynamic interface, BBB’s structure is often damaged in pathological conditions, leading to the disruption of barrier function, which is mainly manifested in the increased permeability of both transcellular and paracellular pathways. Lipopolysaccharide (LPS), also known as endotoxin, which is the cell wall component of Gram-negative bacteria, is the main cause of sepsis (Opal, 2010; Yan et al., 2016; Berger et al., 2017; Hao et al., 2017; Skirecki and Cavaillon, 2019). Purified LPS is widely used to construct pathological models of sepsis and its complications (Kingsley and Bhat, 2016; Varatharaj and Galea, 2017). Intensive researches have demonstrated that LPS could destroy the BBB through a variety of mechanisms, and the damaged BBB would promote the development of multiple brain diseases, including the sepsis-associated encephalopathy (SAE) (Sweeney et al., 2019). In recent years, SAE caused by the disrupted BBB has attracted extensive attention, in which a CNS often has no obvious infection, but patients have significant cognitive dysfunction, memory decline, and other symptoms of serious complications (Iwashyna et al., 2010; Golzari and Mahmoodpoor, 2014; Varatharaj and Galea, 2017; Kikuchi et al., 2019).

In view of the fact that LPS can damage the BBB to cause serious diseases, here we review the relevant literature in recent years to elaborate the specific mechanism of LPS on BBB damage from two aspects of direct and indirect effects, and to discuss the links between BBB damaging by LPS and SAE, and the possible means to treat SAE by targeting the BBB.

## Direct Effects

### Paracellular Pathways

Controlling substances and cells into and out of CNS is the most important function of BBB, especially preventing toxins and pathogens from entering the brain. It guarantees the relative stability of CNS under different conditions, which is conferred by the low permeability of transcellular pathway and paracellular pathway of BBB. The low permeability of paracellular pathways is mainly determined by tight junctions (TJs), which mainly consist of densely distributed transmembrane protein claudin (especially claudin-5), occluding, tricellulins, junctional adhesion molecules, and intracellular support proteins such as zona occludens (ZO) (Haseloff et al., 2015; Tietz and Engelhardt, 2015). TJs are mostly located between brain ECs, sealing the paracellular pathway of molecules, greatly reducing the permeation of polar solutes from plasma to extracellular fluid of the brain, which play an important role in maintaining the integrity of BBB (Sivandzade et al., 2020; Yu et al., 2020; Winkler et al., 2021). In addition, adherens junctions (AJs) formed by intercellular cadherin (e.g., vascular-endothelial (VE-) cadherin) and intracellular catenin (e.g., epithelial (E-)cadherin), which connect cells together, are the basis of TJs and are also involved in maintaining the barrier function of BBB (Blecharz-Lang et al., 2018; Yang et al., 2018).

**Figure 1:**
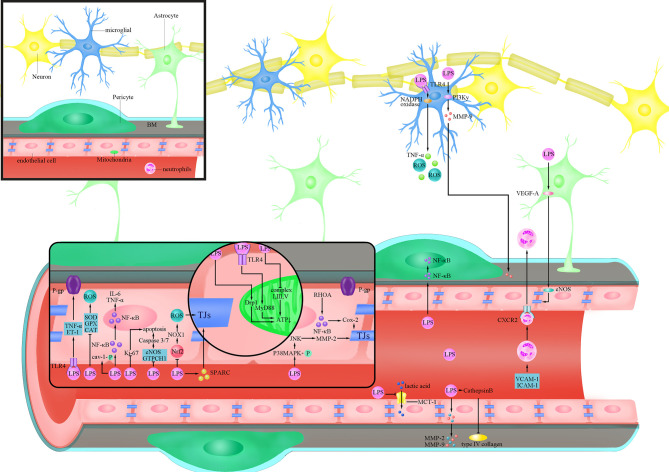
Cellular responses of BBB evoked by LPS. lipopolysaccharide (LPS) activates Ras homolog family member A (RHOA) and Nuclear factor-k-gene binding (NF-κB), and destroys the tight junctions (TJs) protein by triggering the production of cyclooxygenase-2 (Cox-2); LPS inhibits the expression of Nuclear factor-erythroid 2-related factor 2 (Nrf2), thereby increasing the expression of Nicotinamide adenine dinucleotide phosphate (NADPH) oxidase (NOX)1 and destroying the TJs protein;LPS increases the phosphorylation of p38 mitogen-activated protein kinase (MAPK), activates c-Jun amino-terminal kinase (JNK), triggers the expression of matrix metalloproteinase (MMP)-2, and destroys the TJs protein; LPS reduces the expression of TJs protein by inducing secreted protein acidic and rich in cysteine (SPARC); LPS activates Tumour Necrosis Factor-α (TNF-α) and endothelin (ET)-1 after binding to Toll-like receptor 4 (TLR4), and reduces the activity of P-glycoprotein(P-gp); LPS increases the phosphorylation of caveolin-1(cav-1); LPS inhibits endothelial nitric oxide synthase (eNOS) and Guanosine triposphate cyclohydrolase 1 (GTPCH1) and increases the activity of Caspase3/7 to trigger cell apoptosis; LPS trigger cell apoptosis by inhibiting the expression of Ki-67; LPS activates dynamin-related protein-1(Drp1) or activates myeloid differentiation factor 88 (MyD88) after binding to TLR4 to reduce ATP produced by mitochondria; LPS inhibits the production of ATP by inhibiting complex I, III and IV LPS inhibits the expression of Superoxide Dismutase (SOD), glutathione peroxidase (GPX) and catalase (CAT) to increase the level of reactive oxygen species (ROS); LPS trigger the translocation of NF-κB into the nucleus to promote the secretion of inflammatory factors such as Interleukin(IL)-6 and TNF-α; LPS inhibits the expression of Monocarboxylate transporter-1(MCT-1) and inhibits lactic acid influx; LPS trigger the secretion of MMP-2/9 in endothelial cells and directly degrades extracellular matrix (ECM) and digest basement membrane (BM); LPS increases the activity of Cathepsin B and cleave type IV collagen; LPS triggers the translocation of NF-κB to the nucleus in pericytes; LPS trigger the increase of the levels of Vascular cell adhesion molecule-1(VCAM-1) and Intercellular adhesion molecule-1(ICAM-1) in blood vessels, as well as the combination of neutrophils and C-X-C Motif Chemokine Receptor 2 (CXCR2) to trigger the infiltration of the brain; LPS trigger the secretion of vascular endothelial growth factor A (VEGF-A) in astrocytes and destroys TJs protein by activating eNOS; LPS binds to TLR4 on microglia and trigger the production of TNF-α and ROS through the NOX pathway; LPS produces MMP-9 through the phosphoinositide 3-kinase (PI3K)γ pathway and acts on the BM.

LPS has been demonstrated in several studies to enhance BBB permeability by killing TJs and AJs, decreasing their expression, and altering their distribution, allowing hazardous chemicals to pass across the BBB and induce CNS dysfunction (Cheng et al., 2018). Seok et al. have confirmed that LPS can reduce the amount of TJs proteins, ZO-1 and claudin-5, and change their cellular localization by transferring them from the cell membrane to the cytoplasm after 14 days’ treatment (Veszelka et al., 2007; Seok et al., 2013; Banks et al., 2015). LPS produced by *Bacteroides fragilis* has been found to damage the BBB by disrupting intercellular adhesion proteins and cleaving cadherin (Alexandrov et al., 2020). Besides, LPS of *Escherichia coli* O111:B4 treatment directly cause the formation of the paracellular gap by increasing the expression of C-X-C Motif Chemokine Receptor 2 (CXCR2) on brain ECs in a time-dependent manner (4-24 h post LPS injection), which was involved in endothelial actin polymerization and actin stress fiber formation (Wu et al., 2020). Another important factor involved in LPS-mediated damage to TJs is matrix metalloproteinase (MMP), especially MMP-9 (Dal-Pizzol et al., 2013), which participates in the degradation of TJs and thereby elevates BBB permeability (Ren et al., 2015). MMP is regulated by a variety of cytokines on the mitogen-activated protein kinase (MAPK) pathway without overexpression in normal brain tissues (Hong et al., 2005; Malinsky et al., 2013; Yi et al., 2020). Qin et al. found that LPS could increase phosphorylation of p38MAPK and inhibition of p38MAPK could significantly inhibit LPS-induced MMP-2 overexpression at both the mRNA and protein levels, thereby attenuating the effect of LPS on occluding (Qin et al., 2015) (**Figure 1**). It is worth mentioning that c-Jun amino-terminal kinase (JNK) in the MAPK family plays a similar function to p38MAPK (Qin et al., 2015). This suggests that the MAPK family may be involved in the process that LPS disrupts TJs (Qin et al., 2015).

Nuclear factor-k-gene binding (NF-κB) is a key regulator of inflammation-related gene expression in human cells, and LPS can stimulate NF-κB expression by binding to Toll-like receptor 4 (TLR4). (Tiruppathi et al., 2008; Verstrepen et al., 2008; Xu et al., 2021). Hu et al. found that LPS can destroy BBB by reducing the level of occludin through TLR4/NF-κB pathway (Hu et al., 2017). Ras homolog family member A(RHOA), which induces intracellular inflammatory signal transduction and acts as a regulator of the integrity of intercellular junctions, has been found to be involved in promoting disassembly of TJs (Forster, 2008; Yamamoto et al., 2008). Studies have found that LPS of *E. coli* O55:B5 can activate RHOA as well as downstream NF-κB, and lead to increased phosphorylation of myosin light chain (MLC), which ultimately causes a decrease in the expression of TJs proteins, claudin-5 and ZO-1 (He et al., 2011) (**Figure 1**). In addition, studies found that the NF-κβ/cyclooxygenase-2 (Cox-2) pathway is involved in the process of BBB damage by LPS of *E. coli* O111:B4, and inhibition of Cox-2 prevented the LPS-induced decrease in Transendothelial electrical resistance (TEER) and improved the amount of ZO-1, showing a decrease in BBB permeability (Kikuchi et al., 2019) (**Figure 1**). Therefore, NF-κB and its associated molecules play an important role in the process of TJs damage in inflammatory responses mediated by LPS, ultimately disrupting BBB function.

Oxidative stress, which is mainly manifested by excessive production of reactive oxygen species (ROS), occurs widely when the balance between oxidation and antioxidation is broken (Tarafdar and Pula, 2018). Treatment of human brain microvascular endothelial cells (BMECs) with LPS of *E. coli* O111:B4 leads to an abnormal increase in ROS, which further caused a significant decrease in occludin and claudin-5 after 8 hours’ treatment, and a time-dependent decrease in TEER, indicating the damage of BBB (Zhao et al., 2014). In addition to causing direct damage to TJs, ROS induced by LPS of *E. coli* O111:B4 can also activate other molecular pathways such as RHOA/phosphoinositide 3-kinase (PI3K)/protein kinase B(AKT) to reduce the expression of occludin and claudin-5, and cause the reduction of TEER (Schreibelt et al., 2007). Studies have proved that the phosphorylation of MAPK can be inhibited when the ROS production induced by LPS of *E. coli* O111:B4 is inhibited, thus decreasing the phosphorylation of TJs and protecting the integrity of BBB (Deli et al., 2005; Cardoso et al., 2010; Seok et al., 2013). Nicotinamide adenine dinucleotide phosphate (NADPH) oxidase is an important source of ROS and is demonstrated to participate in the process of BBB dysfunction (Chrissobolis and Faraci, 2008; Ma et al., 2017). In the study of Zhao et al., LPS of *E. coli* O111:B4 treatment increased the activity of NADPH oxidase (NOX) in a time-dependent manner (0-24 hours post LPS treatment), and the accumulation of ROS induced by LPS was significantly reduced after inhibiting the activity of NOX (Zhao et al., 2014). Exogenous antioxidants such as Superoxide Dismutase(SOD) and 4,4’-Diaminodiphenylsulfone, Dapsone (DDS) were found to reduce the damage of LPS to BBB by preventing LPS-induced ROS accumulation, thereby restoring the expression of TJ proteins to maintain the low permeability of BBB (Miller et al., 2009; Cho et al., 2010; Zhou et al., 2014).In view of this, activating endogenous antioxidant molecules inhibited by LPS may be a better solution to attenuate the damage of LPS to BBB. As a transcription factor that regulates the expression of multiple antioxidant genes, Nuclear factor-erythroid 2-related factor 2 (Nrf2) deficiency was found to cause an increase in intracellular ROS levels (Hyeon et al., 2013). Li et al. found that LPS treatment led to an increase production of ROS in both *in vivo* and *in vitro* experiments by inhibiting the expression of Nrf2, which was accompanied by decreased TEER and ZO-1, two indicators for a breakdown of the BBB (Li et al., 2018) (**Figure 1**). After artificially silencing the expression of Nrf2, a significant increase in ROS related to the enhanced expression of NOX1 can be observed, and this effect was amplified by the presence of LPS (Li et al., 2018) (**Figure 1**). Researchers have attempted to activate AMPK in both genetic and non-genetic ways since long-term exposure to *E. coli* O111:B4 LPS was shown to cause a reduction in its activity (Zhao et al., 2014; Taile et al., 2020). The results showed that activation of AMPK significantly reduced LPS-induced NOX and ROS production after 24 hours’ treatment, ultimately improving the expression of occludin and claudin-5 (Zhao et al., 2014). Activating AMPK with metformin by Takata et al. showed reduced permeability of BBB, which manifests itself specifically as an increase in TEER and a decrease in Evans blue albumin permeability (Takata et al., 2013). Therefore, LPS can dysregulate the oxidative and antioxidant responses in ECs by inducing the expression of ROS, which ultimately leads to damage of TJs and disruption of BBB.

As a cysteine rich acidic secreted protein, secreted protein acidic and rich in cysteine (SPARC) is highly expressed not only at the early stage of cerebrovascular development, but also at sites such as injury and tumors, and it is related to the angiogenesis and the integrity of BBB (Vincent et al., 2008). Addition of exogenous LPS caused an increase in SPARC, which in turn led to a decrease in the expression of ZO-1 and occluding, and finally a rise in BBB permeability at 24 h in a concentration-dependent manner (Alkabie et al., 2016). Researchers believe that SPARC’s primary function after stimulated by LPS is to promote ECs proliferation at the damaged site and to protect BBB, but SPARC also reduces the expression of ZO-1 and occluding, and eventually increases BBB permeability (Bradshaw and Sage, 2001; Endl et al., 2001; Chlenski and Cohn, 2010; Alkabie et al., 2016) (**Figure 1**).

### Transcellular Pathway

Concerning the transcellular transport of substances by the BBB, it is generally accepted that there are four pathways to transport ions and molecules between the blood and the brain (Daneman and Prat, 2015; Langen et al., 2019): i) Free diffusion of lipid-soluble substances and gas small molecules; ii) polar molecules that cannot diffuse freely are transported by solute carriers (Winkler et al., 2015; Chien et al., 2018); iii) A variety of lipid-soluble substances can be selectively transported by ATP-binding cassette transporters (ABCs) (Bauer et al., 2019); iv) Vesicular transport of macromolecules, also known as transcytosis. Notably, vesicular transport in ECs at the BBB was slower than that in most peripheral ECs, which may be due to inhibition of caveolae-mediated transcytosis (Andreone et al., 2017; Sadeghian et al., 2018). Since the first two pathways is mainly related to the concentration difference of intracellular and extracellular solutes rather than a regulatory role of the cell itself, the low permeability of BBB is achieved primarily by the efflux effects of ABCs and the inefficient vesicle transport.

P-glycoprotein (P-gp), an important efflux transporter belonging to ABCs (Linville et al., 2019). Reduced expression and activity of P-gp may cause an accumulation of toxic metabolites in the CNS, leading to an increased risk of cerebral diseases. Studies showed that LPS of *E. coli* O111:B4 can disrupt BBB permeability by affecting the activity of P-gp, causing various neurotoxins and pathogenic microorganisms to fail to drain out of the brain (Veszelka et al., 2007; Dohgu and Banks, 2008; Cardoso et al., 2012). In the study of Hartz et al., [N-ϵ(4-nitrobenzofurazan-7-yl)-d-Lys8]-cyclosporin A, a substance that accurately and specifically reflects the activity of P-gp in the vascular lumen, was declined in a concentration-dependent way after brain capillaries were treated with LPS, indicating a decrease in the activity of P-gp upon LPS treatment (Hartz et al., 2006). Further studies showed that LPS activates molecules such as tumor necrosis factor-α (TNF-α), endothelin (ET)-1 by binding to TLR4 on the EC membrane, which ultimately causes a rapid and reversible decline in P-gp activity (Bauer et al., 2004; Hartz et al., 2006) (**Figure 1**). In the study of Salkeni et al., they excluded the roles of blood circulating factors in the actions of LPS of *Salmonella typhimurium* on the activity of P-gp by intraperitoneal perfusion of CD-1 mice, and their results also showed that LPS inhibited the activity of P-gp independent of prostaglandins or nitric oxide (NO) (Salkeni et al., 2009). It is worth noting that LPS reduced P-gp activity by directly activating nitric oxide synthase (NOS) by Hartz et al., which is inconsistent with the study by Salkeni et al., and the possible reason may be due to the involvement of microglia in the latter study (Hartz et al., 2006; Salkeni et al., 2009). Pan et al. showed that LPS of *S. typhimurium* regulated NF-κB to elevate P-gp expression after 48 hours’ treatment, while the efflux activity of P-gp decreased, so the regulation of P-gp by LPS was most likely to be achieved by post-translational modifications (Hartz et al., 2006; Pan et al., 2010).

In addition to ABCs, caveolae-associated transcytosis also plays an important role in maintaining low permeability in the transcellular pathway of the BBB. Caveolin-1 (cav-1) is a major component of caveolae and its expression level or activity would have a significant impact on transcellular permeability (Keaney and Campbell, 2015; Liu et al., 2015; Linden et al., 2019). Wang et al. found that LPS had no obvious effect on the expression of cav-1, but would increase the phosphorylation degree of cav-1 after treatment for 4 and 24 hours, and inhibition of cav-1 phosphorylation with drugs was accompanied by a decrease in BBB permeability (Wang et al., 2019a) (**Figure 1**). Increased phosphorylation of cav-1upon LPS treatment may have led to structural changes of caveolae, ultimately elevating the permeability of BBB. Collectively, LPS affects the permeability of the BBB transcellular pathway mainly through regulating of P-gp and cav-1.

### Endothelial Cells

Vascular ECs constitute the main cellular components of the BBB, and structural damage to the endothelium or a decrease in the number of ECs may elevate the permeability of the BBB (Tam and Watts, 2010; Banks et al., 2015). Studies showed that the apoptosis of ECs is closely related to BBB damage and cerebral microcirculation disturbance, so the effect of LPS on the apoptosis of ECs of BBB may refine the mechanism of damage to the BBB by LPS (Yamazaki et al., 2019). Cardoso et al. found that LPS of *E. coli* O111:B4 treatment causes time-dependent apoptosis of mouse BMECs (Cardoso et al., 2012). A similar result was also reported by Li et al., who found that LPS could increase the apoptosis of bend.3 cells and decrease their activity after 24 hours’ treatment (Li et al., 2020). To further clarify the underlying mechanism of cell apoptosis induced by LPS, apoptosis analysis of human microvascular endothelial cell(HCMEC/D3) treated by LPS was performed by Liu et al (Liu et al., 2020). They showed that as the concentration of LPS increased, the activity of Caspase3/7 rose, the expression of the pro-oncogene Bax increased and the tumor suppressor gene Bcl-2 decreased (Liu et al., 2020) (**Figure 1**). They further found that the effects of LPS on HCMECs could be reversed by enhancing the expression of endothelial nitric oxide synthase (eNOS) and Guanosine triphosphate cyclohydrolase 1 (GTPCH1), two proteins that are involved in ECs protection, indicating that LPS may damage the function of BBB by promoting EC apoptosis *via* inhibiting eNOS and GTPCH1 (Zheng et al., 2018; Liu et al., 2020) (**Figure 1**). In addition, Boitsova et al. found that the expression of Ki-67 protein, which is associated with cell proliferation, significantly decreased in rat BMECs after treated the LPS of *E. coli* O111:B4 for 72 hours (Boitsova et al., 2018) (**Figure 1**). These results indicated that LPS can promote apoptosis and reduce the proliferation of EC, and ultimately destroy the structure and disrupt the function of BBB.

Mitochondria are the center of cellular energy metabolism. Abnormal energy metabolism of mitochondria in ECs of the BBB is usually followed by bioenergetics disruption of ECs, which may finally cause the BBB dysfunction (Alelwani et al., 2020). Haileselassie et al. found that mitochondrial damage in ECs and subsequent disruption of the BBB were caused by reduced mitochondrial membrane potential and increased oxidative stress after LPS treatment (Haileselassie et al., 2020). They also showed that the permeability of microvascular ECs/astrocytes co-culture system increased upon LPS treatment for 24 hours, and this phenomenon resulted from pathological activation of dynamin-related protein-1 (Drp1) (Haileselassie et al., 2020)(**Figure 1**). Inhibition of Drp1 with P110, an inhibitor of Drp1- Fission 1 (Fis1) interaction, was found to attenuate LPS induced oxidative stress as well as elevate the membrane potential of mitochondria, which consequently improved the integrity of BBB (Haileselassie et al., 2020). Besides, the injection of LPS resulted in a decline in the mitochondrial ATP as well as a rise in ROS levels in the brain, which might be attributed to LPS of *E. coli* O55:B5 interfering with mitochondrial transcription as well as oxidative phosphorylation (Suliman et al., 2003). Monocarboxylate transporter-1 (MCT-1), as a lactate receptor expressed at luminal and abluminal membranes, carries on the influx and efflux of lactate between cells and contributes to the effective metabolic coupling of NVU cells (Lottes et al., 2015). Boitsova et al. found that LPS of *E. coli* O111:B4 treatment inhibited the expression of MCT-1 in BMECs after 24 hours’ treatment, which consequently leads to insufficient uptake of lactate by cells (Boitsova et al., 2018) (**Figure 1**). Thereby, the mitochondrial dynamic effects mediated by lactate are later suppressed, and then the metabolic state of mitochondria, especially energy production, was disrupted. As lactate transport is closely associated with proliferation activity of BMECs and highly selected permeability of the BBB, it was found that cell proliferation activity decreased while BBB barrier function was impaired in the presence of LPS (Stapor et al., 2014; Tang et al., 2014).

To explore the roles of LPS in oxidative phosphorylation and mitochondrial volume in ECs, cultured cerebrovascular endothelial cells (cCVECs) were used as an *in vitro* BBB model by Doll et al. (2015). They found that the expression of myeloid differentiation factor 88 (MyD88), an adaptor protein involved in TLR4 signaling, increased in cCVECs upon LPS of *E. coli* O55:B5 stimulation, and it was clearly colocalized with mitochondria, suggesting that LPS could affect mitochondrial function directly through TLR4 mediated signaling (Doll et al., 2015; Fritz et al., 2018) (**Figure 1**). They further analyzed the effect of LPS of *E. coli* O55:B5 on mitochondrial energy metabolism by detecting cellular energy consumption oxygen. Results showed that no significant change in basal oxygen consumption was observed, but the maximal respiration and spare capacity in cCVECs decreased significantly, which were not related to the decrease of cell number or cell viability (Doll et al., 2015; Lou et al., 2016). These results demonstrate that the impairment of mitochondrial energy metabolism in ECs occurs after LPS treatment (Doll et al., 2015; Lou et al., 2016). Considering that it is the respiratory chain complex proteins that play a decisive role in mitochondrial respiration, the expression of the respiratory chain complexes was examined after LPS treatment by Doll et al (Doll et al., 2015; Chen et al., 2018; Tsai et al., 2020). They demonstrated that the expression of complex I, complex III, and complex IV decreased after LPS of *E. coli* O55:B5 treatment, suggesting that LPS inhibits oxidative phosphorylation of CVECs (Doll et al., 2015) (**Figure 1**). Specifically inhibiting complex I or V caused a rapid rise in the permeability of cCVEC and a disruption of linear cell-cell junctions in both *in vivo* and *in vitro* experiments (Doll et al., 2015). Therefore, mitochondria in ECs plays a key role in maintaining BBB integrity, and LPS can damage the barrier function of the BBB by inhibiting mitochondria.

The oxidative stress and inflammation in ECs induced by LPS can damage BBB (Zhang et al., 2010; Zhao et al., 2015). Li et al. found that the expression of three antioxidant enzymes, glutathione peroxidase (GPX), catalase (CAT), and SOD were inhibited in Bend.3 cells after LPS treatment for 24 hours, which resulted in an increased level of ROS (Ceretta et al., 2012; Li et al., 2020) (**Figure 1**). In addition, reduced glutathione/oxidized glutathione (GSH/GSSG), an indicator of cellular antioxidant capacity, was similarly inhibited in Bend.3 cells by LPS after 24 hours’ treatment (Buendia et al., 2016; Lu et al., 2016; Ugun-Klusek et al., 2017; Zheng et al., 2021). Li et al. also showed that the activation of Nrf2 elevated the expression of antioxidant enzymes GPX, CAT, SOD as well as GSH/GSSG, which alleviated the BBB dysfunction induced by LPS (Li et al., 2020). These results suggest that inhibition of the antioxidant capacity of ECs leading to a rise in the level of ROS is an important mechanism that LPS causes BBB damage. In addition, mouse BMECs were also found to produce inflammatory responses upon LPS stimulation, specifically by directly producing inflammatory factors such as interleukin (IL)-1β, IL-18, IL-6, and TNF-α, and these factors are able to mediate the damaging effects of LPS on the BBB (Smyth et al., 2018; Li et al., 2020). As oxidative stress and the inflammatory response are frequently inextricably linked, occurring concurrently in response to LPS stimulation and interacting with each other, both aspects need to be taken into consideration to protect ECs and BBB (Zhao et al., 2011; Li et al., 2020).

Based on the above studies we believe that LPS can disturb the functional status of the BBB by directly affecting processes such as proliferation or apoptosis of ECs, mitochondrial energy metabolism, inflammatory responses, as well as oxidative stress.

### Basement Membrane

The composition of BBB, including vascular ECs, pericytes, and the foot processes of astrocytes, are all attached to the BM, which is composed of extracellular matrix (ECM) (Cardoso et al., 2010). Once the BM is disrupted, the function of the BBB will also be impaired. MMP can directly degrade the ECM as well as digest the BM (Cardoso et al., 2010). Studies found an increased expression of MMP upon LPS treatment, which was accompanied by increased permeability of BBB (Tilling et al., 1998; Yang et al., 2007; Vanlaere and Libert, 2009; Cardoso et al., 2010; Coisne and Engelhardt, 2011; Lee et al., 2011; Zhao et al., 2011; Qin et al., 2015).

Dal-Pizzol et al. used cecal ligation and puncture (CLP, a widely used animal model of sepsis without direct injection of LPS), which allows intestinal bacteria to invade the body cavity to establish a sepsis model and found that the increased level of LPS in the blood was accompanied by elevated BBB permeability (Dal-Pizzol et al., 2013). They further showed that the elevated permeability of BBB was most likely to be related to the increased contents and activity of MMP-2 and MMP-9 in the presence of LPS, as the injection of inhibitors of MMP-2 and MMP-9 reduced BBB permeability in model mice (Dal-Pizzol et al., 2013). Cardoso et al. also confirmed that the expression of MMP-2 and MMP-9 in rat BMECs was significantly increased after LPS of *E. coli* O111:B4 treatment for 4 hours, and results of sodium fluorescein and TEER assays showed obviously disrupted barrier function of BMECs (Cardoso et al., 2012) (**Figure 1**). Inhibition of MMP-9 was also found to protect the microvascular BM in the presence of LPS in a study by Wang et al (Wang et al., 2019a). In addition, they discovered that in response to LPS, the activity of cathepsin B, which can cleave type IV collagen, an important component of BM, was significantly increased in plasma and brain tissue, which is consistent with the finding that the distribution of type IV collagen in brain ECs became discontinuous after LPS stimulation by confocal microscopy (Shoji et al., 2014; Daneman and Prat, 2015; Wang et al., 2019a)(**Figure 1**). Pericytes, an essential component of the NVU, wrap around the surface of ECs and embed in BM (Sweeney et al., 2016). Nishioku et al. found that rupture of BM occurred after LPS of *E. coli* O55:B5 treatment and that pericytes are detached from the rupture of BM, which were accompanied by an increase of the permeability of BBB at 6 and 24 hours (Nishioku et al., 2009). Collectively, the integrity of BM is essential for maintaining the barrier function of BBB and the damage to the BM by LPS would lead to the destruction of BBB.

## Indirect Effects

### Microglia

As an integral part of the NVU in the BBB, microglia play an important role in maintaining the integrity of BBB (da Fonseca et al., 2014; Liebner et al., 2018). Activating microglia surround blood vessels is a protective response that prevents more pathogens from entering the brain tissue (Bowyer et al., 2020). Sumi et al. found that a small dose of LPS of *E. coli* O55:B5 could activate most of microglia in the rat brain microvascular endothelial cell (RBEC)/microglial co-culture system at 6 hours, which appeared as a morphological change from round to bipolar rod shape with enlarged cytoplasm and cell bodies, and an enhanced ionized calcium binding adapter molecule 1 (Iba1) staining (Nishioku et al., 2009; Sumi et al., 2010). The activation of microglia by LPS of *E. coli* O55:B5 resulted in reduced RBEC permeability, and the degree of this effect was closely related to the amount as well as the degree of activation of microglia (Nayak et al., 2010). Further immunofluorescence staining showed that due to the activation of microglia, the immunostaining activity of ZO-1, claudin-5, occludin, and other TJ proteins in the intercellular borders became fragmented, and the above dysfunction could be alleviated by NOX inhibitor (Sumi et al., 2010). Considering that NOX is an important source of ROS, and LPS can promote the production of inflammatory factors by binding to TLR4, researchers believe that LPS in microglia destroys the structure and function of BBB by generating ROS through the TLR4-NOX signaling pathway (Chow et al., 1999; Akira and Takeda, 2004; Sumi et al., 2010; Anthoney et al., 2018) (**Figure 1**). Also, Nishioku et al. demonstrated the disruption of BBB by TNF-α only in the presence of microglia (Nishioku et al., 2010) (**Figure 1**). Bowyer et al. found that the microglia can be activated by LPS, which were particularly manifested as a mass migration to blood vessels (Bowyer et al., 2020). This study revealed that LPS activates microglia not only by directly binding to receptors on them, but also by released cytokines by damaging blood vessels.

We previously described the mechanisms that LPS enhance BBB permeability by altering P-gp activity in ECs, and some researchers believe that vascular ECs and microglia are also involved in these processes. Matsumoto et al. used the accumulation of rhodamine 123 in cells to assess the function and activity of P-gp, and discovered that LPS of *E. coli* O55:B5 may decrease P-gp activity in brain ECs in a microglia-dependent way after 6 hours’ treatment (Matsumoto et al., 2012). They further proved that LPS can reduce the activity of P-gp by activating NOX in microglia to produce inflammatory factors such as TNF-α (Matsumoto et al., 2012; Carey et al., 2020). PI3K-γ is a key mediator of LPS induced activation and phagocytosis of microglia. Frister et al. found that in microglia, LPS of *E. coli* O55:B5 increased the expression of MMP in a PI3K-γ-dependent pathway, thereby increasing the permeability of the BBB (Frister et al., 2014) (**Figure 1**). Therefore, like ECs, microglia can also produce MMP to destroy the BM and open BBB (Yadollah et al., 2003). Taken together, microglia participate in damaging the barrier function of BBB in response to LPS through inflammatory cascade amplification and oxidative stress (Desai et al., 2007; Gao and Hong, 2008).

### Astrocytes

As an integral part of the NVU of the BBB, astrocytes attach to capillaries of the CNS as end feet, and secrete various trophic factors and ECM proteins (Kaplan et al., 2020). In response to stimulation with LPS, astrocytes are activated to secrete cytokines, which are followed by activation of microglia, triggering a more drastic inflammatory response (Tarassishin et al., 2014; Vutukuri et al., 2018; Bowyer et al., 2020; Silva et al., 2020). Park et al. found that after male Sprague-Dawley rats were injected with LPS, the filaments and terminal feet of astrocytes were significantly sparse at 12 hours and 7 days, and these changes were accompanied by increased vascular permeability, which was assessed by infusing the vessel and then visualizing the concentration of extravasated vessels, suggesting that the integrity of BBB was disrupted (Park et al., 2015). Studies have further shown that LPS elevates the expression of vascular endothelial growth factor A (VEGF-A) by astrocytes, followed by activating eNOS, inhibiting the expression of claudin-5 and occluding, and ultimately disrupting the barrier function of the BBB (Argaw et al., 2009; Argaw et al., 2012; Park et al., 2015) (**Figure 1**). Therefore, maintaining the normal morphology and function of astrocytes is an important way to preserve BBB function.

### Pericytes

It is now widely recognized that pericytes are spatially the closest cells to brain ECs, and the roles of pericytes in the process of LPS damaging BBB have attracted much attention (Bonkowski et al., 2011; Mae et al., 2011). Vutukuri et al. found that the activation of pericytes was observed in whole male C57BL/6 mice brain tissue after LPS of *E. coli* O55:B5 treatment for 4 hours, which was accompanied by an increase in the levels of TNF-α and IL-1β, and in the permeability of BBB (Vutukuri et al., 2018). Smyth et al. found translocation of NF-κB into the nucleus in pericytes after LPS of *E. coli* O26:B6 treatment for 1 hour, indicating an activated inflammatory response in pericytes in the presence of LPS (Smyth et al., 2018) (**Figure 1**). Further studies have found that the inflammatory response mediated by signal transducer and activator of transcription 1(STAT1), and similar to others against dodecapentaplegic-2/3(SMAD2/3) is also very similar in pericytes and ECs (Smyth et al., 2018). These results suggest that inflammatory responses mediated by pericytes and ECs have a concerted response (Smyth et al., 2018). Kovac et al. found that LPS of *S. typhimurium* can stimulate pericytes to produce many chemokines and pro-inflammatory factors (Kovac et al., 2011). Notably, IL-9 generated by pericytes in response to LPS of *S. typhimurium* can further stimulate astrocytes to produce chemokine-ligand-20 (CCL-20), which promotes T lymphocyte infiltration into the CNS and amplifies the inflammatory response *in vivo* (Zhou et al., 2011). Pericytes may therefore play a role in the inflammatory response in the brain tissue upon LPS stimulation to disrupt the barrier function of BBB.

### Neutrophils

The BBB prevents peripheral immune cells from entering the brain tissue, but when the BBB barrier function is broken, they can penetrate the brain tissue, highlighting that the systemic inflammatory response may compromise the BBB’s barrier function (Obermeier et al., 2013; Prinz and Priller, 2017). Park et al. found that neutrophils were infiltrated in brain tissue in male Sprague–Dawley rats after treated with LPS for 12 hours and 7 days, and BBB destruction occurred simultaneously (Park et al., 2015). Haileselassie et al. showed that vascular leukocyte adhesion factors such as vascular cell adhesion molecule-1(VCAM-1) and intercellular adhesion molecule-1(ICAM-1) increased in LPS model of sepsis after 24 hours’ treatment, indicating that inflammatory cells have adhered and transmigrated to vessels (Haileselassie et al., 2020) (**Figure 1**). The CXCR2, a G protein coupled receptor, is involved in a series of leukocyte cellular responses, such as cell recruitment and migration (**Figure 1**) (Jones et al., 1997; Casilli et al., 2005). Wu et al. found that neutrophils were significantly infiltrated in the cerebral cortex of mice after LPS of *E. coli* O111:B4 injection for 24 hours, while blocking CXCR2 significantly alleviated the effects (Wu et al., 2020) (**Figure 1**). Silwedel et al. also observed that LPS of *E. coli* O55:B5 caused an increased expression of C-X-C motif chemokine ligand (CXCL)11 and a decreased level of CXCL12 after 4 hours’ treatment, which resulted from the increase of leukocyte migration (Silwedel et al., 2018). In summary, LPS can induce the migration of neutrophils to the brain tissue. However, studies found that LPS of *E. coli* O111:B4 elevates CXCR2 on brain ECs but reduces CXCR2 on neutrophils, suggesting that ECs are important in inducing neutrophil infiltration under the stimulation of LPS (Rios-Santos et al., 2007; Wu et al., 2020). As an important part of systemic inflammation, the response of neutrophils is specifically manifested as mediating and amplifying inflammation in the brain tissue, thereby destroying the structure of the BBB.

## BBB and SAE

Sepsis, a pathological process causing dysfunction of one’s own organs due to bacterial infection and its resulting dysregulated inflammatory response, is the leading cause of death in critically ill patients with significant morbidity and mortality, representing a high proportion of readmissions after treatment discharge (Fleischmann et al., 2016a; Fleischmann et al., 2016b; Chen et al., 2019; Gadre et al., 2019). Sepsis often leads to multiple organ dysfunctions, such as the heart, liver as well as kidneys, and causes severe complications (Savio et al., 2017; Sun et al., 2018; Poston and Koyner, 2019). The brain dysfunction caused by sepsis, namely SAE, is one of the most common and serious complications (Annane and Sharshar, 2015; Savio et al., 2017; Sun et al., 2018; Ziesmann and Marshall, 2018; Poston and Koyner, 2019; Loscher and Gericke, 2020). The CNS of patients with SAE often does not have obvious infections, instead neuroinflammation and oxidative stress appear in brain tissue, and patients have significant symptoms such as cognitive dysfunction, memory decline, and clinical coma or confusion (Iwashyna et al., 2010; Gofton and Young, 2012; Golzari and Mahmoodpoor, 2014; Catalao et al., 2017; Jesus et al., 2020). Given that disruption of the BBB is an important mechanism underlying the development of SAE and would confer a poor prognosis and long-term impact on quality of life, we will focus on the relationship between BBB destruction and SAE.

### BBB Disruption and SAE

It is commonly assumed that neuroinflammation produced by oxidative stress and mitochondrial dysfunction are the primary pathophysiological processes implicated in the development of SAE. (Cornelius et al., 2013; Sonneville et al., 2013). In normal circumstances, only a small number of inflammatory factors such as TNF-α, IL-1, and IL-6, from the systemic circulation may pass through the BBB to enter the brain (Tauber et al., 2017). However, the disruption of BBB will make a wide range of inflammatory cytokines, neurotoxins, complement as well as pathogens to enter the brain tissue when systemic inflammation occurs (Dhaya et al., 2018; Nwafor et al., 2019; Ren et al., 2020). Not only that, the NVU that makes up the BBB also mediates partial inflammatory responses, oxidative stress, and other reactions, for example, activating microglia to release NO, cytokines, and ROS, to aggravate BBB dysfunction in turn, so BBB disruption can be not only a cause but also a consequence of SAE (da Fonseca et al., 2014; Danielski et al., 2018).

Alexander et al. found that after intraperitoneal injection of LPS of *E. coli* O55:B55, the concentration of TNF-α, a key metabolic mediator in sepsis in the blood, increased significantly after 1 hour’s treatment, which resulted in a decrease in the integrity of BBB, infiltration of neutrophils into the brain and the subsequent SAE symptoms (Alexander et al., 2008). An increased activation of glial cells, as well as neuronal apoptosis, was observed over time by using LPS of *E. coli* O127:B8 to induce peripheral inflammatory responses by Semmler et al (Semmler et al., 2005). In a sepsis model with CLP, Imamura et al. found that the LPS level in the blood increased, the BBB was destroyed, the expression of IL-1β and its receptors in the brain tissue increased, and brain inflammation occurred (Imamura et al., 2011). They also showed that IL-1β was one of the first increased inflammatory factors in sepsis and the dysfunctional BBB, in turn, allowed other inflammatory mediators including IL-1β to enter the brain as well as the cerebrospinal fluid, consequently leading to the appearance of cognitive impairment (Imamura et al., 2011; Mina et al., 2014). Mina et al. used IL-1β receptor antagonist to antagonize IL-1β, which reversed BBB damage and cognitive impairment after brain oxidative damage, confirming that IL-1β is an important factor mediating cognitive impairment in sepsis (Mina et al., 2014).

It is known that during the early stage of sepsis, there is an activation of glial cells at the brain vessels as well as the secretion of large amounts of cytokines, which leads to the disrupted BBB and symptoms of brain inflammation (Comim et al., 2011). Microglia are brain resident macrophages that are normally in a resting state and can immediately go into an activated state upon stimulation by injury or factors like LPS, after which they rapidly undergo proliferation, migration, engulf and secrete cytokines (Greenhalgh et al., 2020; Zengeler and Lukens, 2021). Normal microglial responses in brain tissue are obligatory for phagocytic clearance of damaged neural cells and tissue debris, but persistent or vigorous microglial responses can damage the CNS (Greenhalgh et al., 2020; Zengeler and Lukens, 2021). When BBB permeability rises, especially in the hippocampus, a large number of cytokines, such as TNF-α and IL-6, enter the brain tissue to cause oxidative stress and inflammatory responses in microglia, which plays a major role in the development of cognitive impairment in SAE (Michels et al., 2015b). Moreover, the oxidative stress response mediated by NOX in microglia manifesting as excessive ROS production, is also involved in the process of LPS damage to TJ and AJ proteins and SAE occurrence (Hernandes et al., 2014). Collectively, microglia can be activated by LPS or cytokines such as TNF-α and IL-6, and destroy the BBB during the initial phase of sepsis by directly mediating neuroinflammation in the brain and participating in the direct spread of neuroinflammation in the course of SAE (Hawkins and Davis, 2005; Ronaldson and Davis, 2020).

Sepsis is a dysregulated systemic inflammatory response that is initially aimed at eradicating pathogens or pathogenic factors, but the abnormal inflammatory response would cause BBB disruption and neuronal damage, accompanied by the appearance of cognitive dysfunction, mental disorder, and other clinical symptoms. Clinical studies demonstrated BBB damage in patients who died of sepsis, and a significant decrease of TJ proteins (Sharshar et al., 2007; Erikson et al., 2020). Sharshar et al. demonstrated that brain damage caused by sepsis was specifically manifested as an elevated permeability of BBB by magnetic resonance imaging, which is strongly associated with poor prognosis (Sharshar et al., 2007). Disrupted BBB with a rise in permeability was also observed in a pathological model of sepsis through CLP (Zhao et al., 2015; Silva et al., 2020). These results indicated that impaired BBB may serve as an initiating link as well as a driver of SAE, which leads to an infiltration of various cytokines and leukocytes in brain tissue and causes neuronal apoptosis and dysfunction, ending up with a series of neuropsychiatric symptoms with a high mortality.

### The BBB Is a Validated Target for the Treatment of SAE

Sepsis is an abnormal or dysregulated host response to stimuli from external factors as well as internal organ dysfunction (Lelubre and Vincent, 2018). Current studies have redefined sepsis as ‘life-threatening organ dysfunction caused by a dysregulated host response to infection’ (Singer et al., 2016).To date, sepsis has been a major public health problem worldwide and a leading cause of the death of critical illness (Vincent et al., 2014; Fleischmann et al., 2016a). Survivors of sepsis usually have physical and cognitive dysfunctions, giving rise to a large burden on society and patients’ families (Iwashyna et al., 2010; Iwashyna et al., 2012). SAE, as one of the most serious and frequent complications of sepsis, leads to an extremely poor prognosis for patients as well as a substantial reduction patient’s life quality. Here, we will discuss the possibility of BBB as an effective target for treating SAE. As LPS can disrupt the structure and function of the BBB, and the damaged BBB is closely related to the development of SAE, we believe that targeting the BBB may be an effective strategy to prevent and treat SAE.

**Figure 2:**
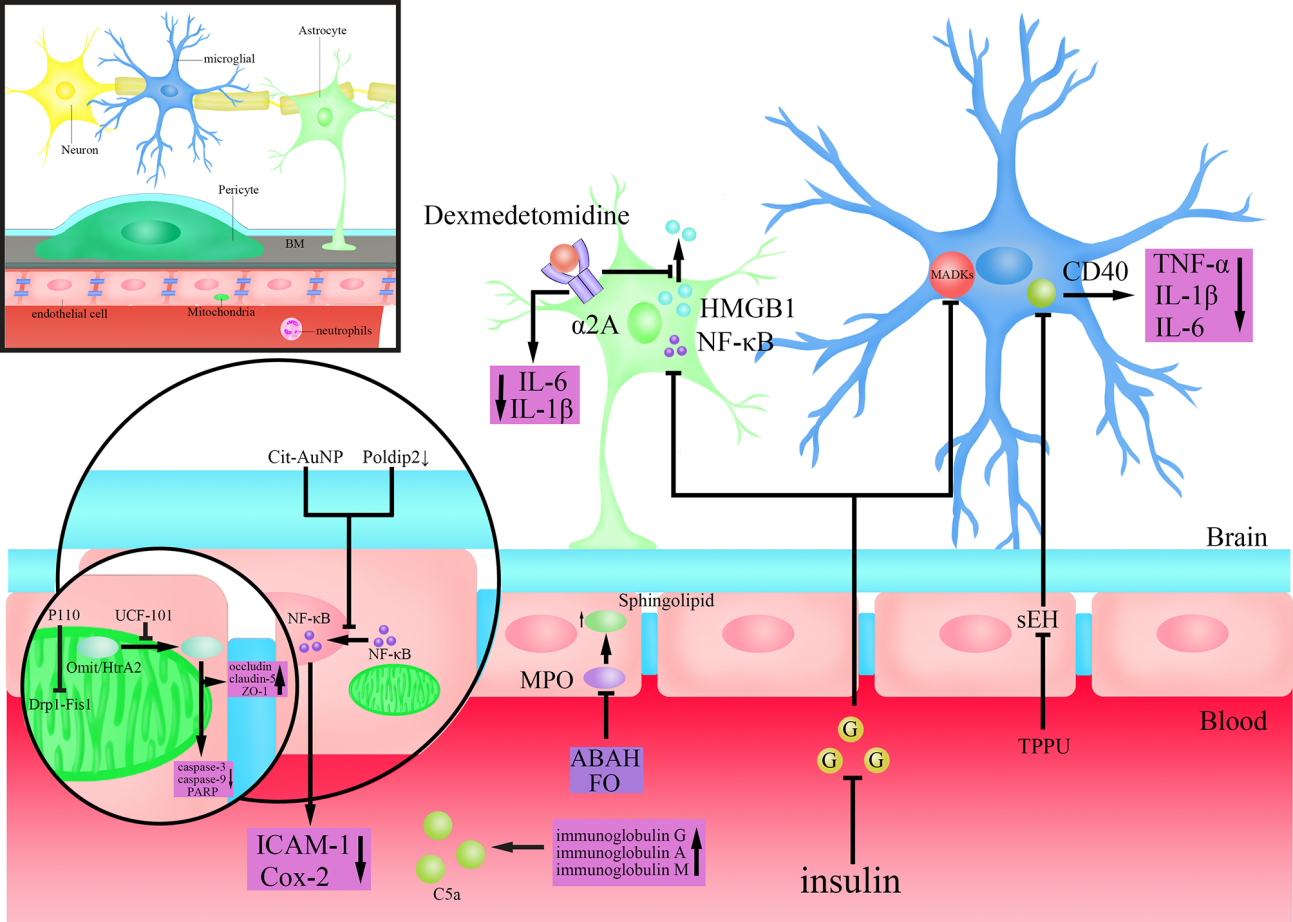
Mechanisms of action of drugs that may target the BBB to treat SAE. Dexmedetomidine activates α2A adrenoceptors on astrocytes to inhibit the extracellular release of high mobility group box protein 1 (HMGB1) and reduce the levels of Interleukin(IL)-6 and IL-1β in brain tissue; Insulin inhibits the activity of Nuclear factor-k-gene binding (NF-κB) in astrocytes and mitogen-activated protein kinase (MAPK)s in microglia after lowering blood sugar; Citrate-covered gold nanoparticles (Cit-AuNP) or targeted inhibition of Poldip2 inhibits the nuclear translocation process of Nuclear factor-k-gene binding (NF-κB) in endothelial cells to reduce the secretion of Intercellular adhesion molecule-1(ICAM-1) and cyclooxygenase-2 (Cox-2); TPPU(N-[1-(1-oxopropyl)-4-piperidinyl]-N’-[4-(trifluoromethoxy)phenyl)-urea) inhibits Cluster of differentiation (CD)40 by inhibiting soluble epoxide hydrolase (SEH), and reduces the secretion of Tumour Necrosis Factor-α (TNF-α), IL-1β and IL-6; 4-aminobenzoic acid hydrazide (ABAH) or Fish oil (FO) maintain the steady state of Sphingolipid by inhibiting Myeloperoxidase (MPO); P110(an inhibitor of dynamin-related protein-1(Drp1)-Fission 1(Fis1) interaction) inhibits the Drp1-Fis1 interaction; UCF-101(an inhibitor of Omi/HtrA2) inhibits the translocation of Omi/high-temperature requirement serine protease A2 (HtrA2) from mitochondria to cytoplasm, antagonizes the Caspase-dependent apoptosis pathway and reduces poly (ADP-ribose) polymerase (PARP) levels, while also protecting the tight junctions (TJs) protein; Injection of immunoglobulin G or a combination of immunoglobulin A and M inhibits complement(C)5a.

Glial cells play an important role in both LPS disruption of the BBB and the development of SAE after BBB disruption (Haruwaka et al., 2019; Spampinato et al., 2019). Monique et al. showed that inhibition of the activity of microglia could decrease the permeability of BBB that were elevated by the actions of systemic cytokines, and reverse the long-term cognitive impairment (Michels et al., 2015b). They further found that the protective effect by inhibiting microglia on BBB is partly through the cluster of differentiation (CD)40-CD40 ligand pathway, and anti-CD40 treatment ameliorated long-term cognitive impairment caused by sepsis (Michels et al., 2015a) (**Figure 2**). Studies in recent years have found that dexmedetomidine, a clinical drug commonly used for sedation, plays a neuroprotective role through an anti-inflammatory mechanism (Liu et al., 2017; Wang et al., 2019b). Mei et al. found that dexmedetomidine could activate 2A adrenoceptors on astrocytes but not microglia to decrease proinflammatory factors in the brain, preserve BBB integrity, and enhance learning and memory performance by activating 2A adrenoceptors on astrocytes in septic mice (Mei et al., 2021) (**Figure 2**). It was shown that the high mobility group box protein 1 (HMGB1) released from astrocytes and microglia is an important factor in regulating BBB permeability in inflammatory response (Festoff et al., 2016) (**Figure 2**). Dexmedetomidine can improve the integrity of BBB as well as cognitive impairment by inhibiting HMGB1 (Hu et al., 2018; Mei et al., 2021). Therefore, HMGB1 may represent a new target for the treatment of sepsis and SAE (Wang et al., 2014). Several studies have demonstrated the pro-inflammatory effects of hyperglycemia (Amorim et al., 2019). Huang et al. found that the activity of astrocytes and microglia, as well as that of NF-κB and MAPKs was inhibited after using insulin to control blood glucose in Sprague-Dawley rats, which accompanied by reduced permeability of BBB and alleviated SAE, showing full recovery of the mechanical withdrawal threshold and thermal withdrawal latency as well as an increased electroencephalography frequency (Huang et al., 2020a) (**Figure 2**). Besides, studies have also confirmed that insulin can directly inhibit brain tissue inflammatory response and oxidative stress injury, which consequently improves brain tissue damage (Chen et al., 2014). Omarigliptin is a novel once-weekly dipeptidyl peptidase-4 (DPP-4) inhibitor developed for the treatment of type 2 diabetes (Biftu et al., 2014). Studies have found that Omarigliptin can not only maintain the integrity of the BBB by inhibiting neuroinflammation, but also reduce the expression of MMP-2 and MMP-9 to protect TJs protein (Du and Wang, 2020). These results suggest that, for the management of sepsis patients in intensive care unit (ICU), attentions should also be paid to the blood glucose changes and the application of insulin, to reduce the likelihood of SAE (Huang et al., 2020a). Overall, protecting the BBB by inhibiting the activity of glial cells may be helpful for the treatment of SAE. But studies also have found that in the early stage of SAE, the morphology of glial cells begins to change without obvious disruption of the BBB (Griton et al., 2020). Therefore, introducing prophylactic drugs that inhibit neuroinflammation would be helpful to avoid the rapid deterioration of SAE after BBB disruption.

NF-κB, as a proinflammatory cell transcription factor that exists in almost all cells, was found to participate in the BBB disruption by LPS (Xu et al., 2021). Nod-like receptor protein 3 (NLRP3) plays an important role in the inflammation cascade by amplifying the inflammation response (Islam et al., 2020). Chen et al. found a suppression of the NF-kB/NLRP3 inflammatory pathway by directly binding to the promoter region of NLRP3, which resulted in a decreased cell apoptosis and inflammation, and an alleviation of BBB disruption and SAE (Chen et al., 2020). Kikuchi et al. showed that inhibition of poldip2 could attenuate BBB disruption caused by sepsis by regulating the nuclear translocation process of NF-κB as well as Cox-2 expression, preventing the occurrence and development of SAE (Kikuchi et al., 2019) (**Figure 2**). Di Bella et al. found that citrate-covered gold nanoparticles (Cit-AuNP) could prevent the process of NF-κB translocation to the nucleus by decreasing the phosphorylation of inhibitor of kappa B alpha (IκBα), which was followed by decreased expression of pro-inflammatory factor ICAM-1 on leukocytes and reduced adhesion of leukocyte to the endothelium of intracerebral blood vessels, ultimately alleviating the inflammatory response in the brain by BBB protection and reducing the occurrence of SAE during sepsis (Di Bella et al., 2021) (**Figure 2**). Wang et al. found that by inhibiting soluble epoxide hydrolase (sEH) using TPPU(N-[1-(1-oxopropyl)-4-piperidinyl]-N’-[4-(trifluoromethoxy)phenyl)-urea), which is required for the activity of epoxyeicosatrienoic acids (EETs), a substance involved in maintaining the intact structure of cerebral blood vessels, the barrier function of BBB was improved, followed by an alleviation of long-term encephalopathy in CLP model mice (Iliff et al., 2010; Wang et al., 2020) (**Figure 2**). Myeloperoxidase (MPO) in microglia and neutrophils not only regulates inflammation-related signaling pathways but also promotes ROS production to mediate oxidative stress (Patel, 2016). Sphingolipid, as the biologically active family of lipids found, plays a key role in maintaining the structure and function of the BBB, and is also closely related to the pathological state of the CNS (Kuperberg and Wadgaonkar, 2017). Ullen et al. found that 4-aminobenzoic acid hydrazide (ABAH), an inhibitor of MPO, alleviated BBB dysfunction induced by LPS by maintaining the homeostasis of sphingolipid, thereby improving the function of brain (Ullen et al., 2013; Goeritzer et al., 2020) (**Figure 2**). Fish oil (FO) is an important source of long-chain n-3 polyunsaturated fatty acids (PUFAs) with immunomodulatory and anti-inflammatory effects (Li et al., 2014). Recent studies have found that FO enriched lipid emulsion is able to prevent long-term cognitive impairment caused by sepsis, by protecting the BBB as well as inhibiting the activity of MPO (Della Giustina et al., 2020) (**Figure 2**). Interestingly, Margotti et al. found that neutrophils infiltration into brain tissue due to BBB rupture caused an increased production of MPO, accompanied by lipid oxidative damage in the hippocampus and prefrontal cortex, suggesting a vicious circle between BBB destruction and MPO production (Margotti et al., 2020). These results suggested that MPO may become a potential drug target for the treatment of brain dysfunction caused by sepsis (Margotti et al., 2020). Besides, anti-inflammatory and antioxidant drugs, such as metformin, platonin have been shown to exhibit neuroprotective effects, and can improve the decreased expression of TJ proteins induced by sepsis, thereby improving BBB function and attenuating sepsis-induced brain injury (Yeh et al., 2015; Ismail Hassan et al., 2020). Therefore, anti-inflammation and anti-oxidation represent important strategies to protect BBB and brain tissues.

Mitochondria are vital to cell metabolism because they provide ATP for living activities *via* oxidative phosphorylation, and play an important part in the cell apoptosis and programmed cell death processes (Bock and Tait, 2020). Impairment of mitochondrial energy metabolism or abnormal apoptosis in BMECs both affect the barrier function of BBB (Chen et al., 2017). Therefore, mitochondria may represent a potential strategy in the treatment of SAE. Haileselassie et al. found that the mitochondrial dysfunction mediated by the interaction of Drp1-Fis1 in response to LPS leads to increased permeability of the BBB and a direct toxic effect on primary cortical ECs, but the above results can all be eliminated by Drp1-Fis1 interaction inhibitor P110 (Haileselassie et al., 2020) (**Figure 2**). So, inhibiting mitochondrial dysfunction to protect both BBB and neurons could be a potentially new therapeutic strategy for treating SAE. Omi/high-temperature requirement serine protease A2 (HtrA2), a proapoptotic serine protease mainly found in and released from mitochondria, is found to be involved in the development of BBB dysfunction and SAE induced by sepsis through a caspase-dependent mitochondrial pathway (Wang et al., 2018; Hu et al., 2019). After inhibiting Omi/HtrA2 translocation from mitochondria to the cytosol by UCF-101(an inhibitor of Omi/HtrA2), oxidative damage in the brain tissue and cognitive dysfunction induced by sepsis were significantly improved (Hu et al., 2013) (**Figure 2**). Further studies found that inhibiting Omi/HtrA2 not only protects neurons, but also alleviates the apoptosis of brain ECs and the decreased TJs protein induced by LPS (Wang et al., 2018; Hu et al., 2019) (**Figure 2**). In addition, Xu et al. alleviated BBB disruption induced by sepsis and protected synaptic plasticity by inhibiting caspase-1-mediated apoptosis, thus improving the cognitive impairment in a mouse model of sepsis (Xu et al., 2019). Therefore, improving mitochondrial dysfunction and related apoptosis pathways are helpful to protect neurons and maintain the barrier function of BBB, which represent a new potential treatment strategy for SAE.

Pathogenic factors in sepsis such as LPS, after binding to TLR4, result in dysregulated inflammatory responses through complex immune activation, so targeting immune activating responses that inhibit neuroinflammation may be protective against BBB disruption by sepsis (Hartz et al., 2006; Miao et al., 2021). Intravenous immunoglobulin is a therapeutic treatment with anti-infective, anti-inflammatory, and immunomodulatory effects (Hamano et al., 2013; Di Rosa et al., 2014). Studies showed that intravenous immunoglobulin can serve as an important part of the adjuvant treatment of sepsis, but for 40 years the method is still controversial (Hamano et al., 2013; Di Rosa et al., 2014; Szakmany, 2019; Jarczak et al., 2020). Esen et al. found that the injection of immunoglobulin G or a combination of immunoglobulin A and M both exerted neuroprotective effects, which improved the integrity of the BBB, and alleviated the symptoms of sepsis in rats (Esen et al., 2012) (**Figure 2**). Considering the regulatory effects of immunoglobulins on the complement system and the finding that inhibition of complement C5a could protect the BBB during sepsis, the protective effect of immunoglobulins on the BBB is most likely achieved through the regulation of the complement system (Basta, 2008; Flierl et al., 2009; Esen et al., 2017) (**Figure 2**). Overall, as the dysregulated inflammatory response in sepsis is coupled with the initial immunomodulatory response, more studies could be performed to refine novel strategies to treat SAE by suppressing the immune activation response of excessive neuroinflammation.

## Conclusion and Future Directions

The BBB is essential for maintaining homeostasis within the brain’s biochemical environment (Liebner et al., 2018). As the barrier of CNS, the low permeability of BBB separates neural tissue from circulating blood, and the infiltration of leukocytes (such as neutrophils, lymphocytes) in CNS is much less than that in peripheral tissues, CNS was often considered as an immune privileged site in the past (Liebner et al., 2018). However, BBB is often disturbed in response to various pathogenic agents, causing the entry of cytokines, pathogens as well as immune cells from blood into the CNS through BBB, which is closely associated with the occurrence and development of CNS diseases such as SAE (Sweeney et al., 2019).

This review discussed the mechanisms of the BBB damage by LPS, a major pathogenic agent of sepsis, through direct and indirect actions. The direct damage on the BBB by LPS is by disrupting the BBB’s structure. Among the mechanisms, the paracellular pathways maintained by TJs and AJs, the transcellular pathway, P-gp, and vesicle trafficking are the fundamental forms by which LPS raises the permeability of the BBB and disrupts its barrier function. The paracellular and transcellular pathways can be regarded as central links in future studies on how to protect the BBB from being damaged by toxic substances like LPS. As the basic components of BBB, the endothelium and BM, are the targets of LPS. Once their energy metabolism status is abnormally altered, a direct disruption of the BBB’s structure would occur and thus its barrier function cannot be maintained. Besides, LPS also damages the BBB indirectly by acting on microglia, astrocytes, pericytes, and neutrophils. Notably, the roles performed by these cells in the disruption of the BBB by LPS may be interpreted as a succession or amplification, implying that the effects on the structure and function of the BBB by LPS are largely or even entirely through the mechanisms detailed in the direct actions section. Recent studies have found that the communication between cells that make up the NVU and the basic structure of the BBB was more complex and diverse than previously realized, and cells of the NVU were involved in dynamically regulating the barrier function of the BBB (Kaplan et al., 2020). Therefore, more researches are needed to explore how to raise the infiltration of drugs into the brain to act on these cells, and thus to protect the BBB.

Sepsis attracts the attention of clinicians and researchers due to the poor prognosis of patients in critical care medicine (Fleischmann et al., 2016a; Fleischmann et al., 2016b; Gadre et al., 2019). In this review, we discussed the link between the BBB disruption and the occurrence of SAE, aiming at finding novel strategies to treat SAE by maintaining the barrier function of BBB. Systemic injection of LPS has been shown to cause damage to the BBB of recipient animals, resulting in the subsequent entry of peripheral cytokines into the brain (Vuuren et al., 2020). It is believed that the main contribution of the damaged BBB to the occurrence and development of SAE is to cause a large number of inflammatory factors and neurotoxins to enter the brain tissue, accompanied by immune cells in the brain tissue such as microglia being activated during systemic inflammation, thereby mediating neuroinflammation and oxidative stress. Subsequently, inflammation and oxidative stress in the brain tissue, in turn, amplify the extent of BBB disruption, which creates a vicious cycle that would fuel the generation and progression of SAE. Therefore, restoring the barrier function of BBB may serve as an effective approach to treat SAE.

According to current research, neuroinflammation and oxidative stress are key factors in the development of SAE, and inhibiting inflammatory reactions has a significant impact on SAE therapy. (Yeh et al., 2015; Kikuchi et al., 2019; Ismail Hassan et al., 2020; Wang et al., 2020; Di Bella et al., 2021). Therefore, elucidating mechanisms of the inflammation and oxidative stress that are closely related to the occurrence of SAE will be important for the development of drugs to treat SAE. More importantly, it was found that drugs that are generally recognized to have the ability to inhibit inflammatory and oxidative stress responses also have neuroprotective effect (Yeh et al., 2015; Ismail Hassan et al., 2020). It is important to note that the efficacy of intravenous immunoglobulin therapy for sepsis has been controversial, but targeting the pathway that inhibits the immune activation response to neuroinflammation is hoped to lead the way from the very beginning to the development of SAE and is likely turn out to be a brand-new strategy in the treatment of SAE. We believe that researchers may try to block the damage on the BBB by LPS to prevent and treat SAE in the further studies.

## Author Contributions

XP and YL conceived and wrote the manuscript. SH and ZL conceived the figure. YL and LZ reviewed the original draft. All authors contributed to the article and approved the submitted version.

## Funding

This study was funded by Innovation and Entrepreneurship Training Program of Sichuan province (S202010632272).

## Conflict of Interest

The authors declare that the research was conducted in the absence of any commercial or financial relationships that could be construed as a potential conflict of interest.

## Publisher’s Note

All claims expressed in this article are solely those of the authors and do not necessarily represent those of their affiliated organizations, or those of the publisher, the editors and the reviewers. Any product that may be evaluated in this article, or claim that may be made by its manufacturer, is not guaranteed or endorsed by the publisher.
